# Lactic Acid Drives ESM1 to Attenuate DNA Damage and CD8+ T Cell Infiltration in Cancer

**DOI:** 10.32604/or.2026.071536

**Published:** 2026-03-23

**Authors:** Yingzheng Tan, Jiao Xiao, Liyun Tang, Jian Wan, Tian Zeng, Wenchao Zhou, Xueru Liu, Xun Chen, Yukun Li

**Affiliations:** 1 Department of Infectious Disease, Zhuzhou Hospital Affiliated to Xiangya School of Medicine, Central South University, Zhuzhou, China; 2 Affiliated Nanhua Hospital, University of South China, Hengyang, China; 3 Tumor ImmunoMetabolism Institute (TIMI), The Affiliated Zhuzhou Hospital Xiangya Medical College, Central South University, Zhuzhou, China; 4 Department of Medical Oncology, The Third People’s Hospital of Yongzhou, Yongzhou, China; 5 Department of Hepatological Surgery, Zhuzhou Hospital Affiliated to Xiangya School of Medicine, Central South University, Zhuzhou, China

**Keywords:** Endothelial cell-specific molecule 1, DNA damage response, lactic acid, CD8 T cell, cancer immunity

## Abstract

**Background:**

Lactate, as a critical byproduct of tumor metabolic reprogramming, plays an important role in DNA damage repair and tumor immune infiltration. This work aims to elucidate the molecular mechanisms by which lactate promotes tumor DNA damage repair (DDR) and subsequent immune evasion.

**Methods:**

Hepatocellular carcinoma (HCC), lung adenocarcinoma (LUAD), and ovarian cancer (OC) cells with cisplatin-induced DNA damage were treated with lactate at a concentration gradient, Endothelial cell-specific molecule 1 (ESM1) shRNA, ESM1 overexpression plasmid, or the Protein Kinase B (AKT) Serine/Threonine Kinase 1 (Akt1) inhibitor LY294002. Proliferation, apoptosis, and DNA damage levels were assessed using 5-ethynyl-2^′^-deoxyuridine (EdU) staining, flow cytometry-based apoptosis assay, and comet assay. Western blot (WB), Polymerase Chain Reaction (PCR), and immunofluorescence (IF) were employed to evaluate the effects of lactate on the expression of ESM1, Akt1, and Cyclic GMP-AMP Synthase (cGAS) pathway-related proteins in cancer cells. Xenograft tumor models were established using ESM1 whole-gene knockout mice, and Cluster of Differentiation 8 Positive (CD8+) T cell infiltration and apoptotic levels in tumors were detected via flow cytometry. Immunohistochemistry (IHC) was performed to examine the expression of ESM1, double-stranded DNA (dsDNA), and CD8 in tumor patient samples, followed by correlation analysis.

**Results:**

This study demonstrates that lactate increases ESM1 mRNA and protein expression in a concentration-dependent manner and reduces DNA damage in tumor cells. Lactate suppresses DDR by activating the Akt1 signaling pathway via ESM1 and further inhibits the cGAS pathway, thereby downregulating the transcription of chemokines and pro-inflammatory factors. *In vivo* experiments confirm that ESM1 knockout promotes CD8+ T cell infiltration into tumors and induces apoptosis. Analysis of tumor patient samples further validates the negative correlation between ESM1 and CD8+ T cell levels in cancer patients.

**Conclusion:**

In summary, lactate activates the Akt1-Murine Double Minute 2 (MDM2)-p53 pathway via ESM1 to suppress DDR, while the reduction of DDR-generated dsDNA inactivates the cyclic GMP-AMP synthase–Stimulator of Interferon Genes (cGAS-STING) pathway, thereby inhibiting CD8+ T cell immune infiltration.

## 1 Introduction

Lactic acid, a critical byproduct of metabolic reprogramming in solid tumors under hypoxic microenvironments, plays a pivotal role in tumor immunity [1]. Recent studies have highlighted that lactate modulates gene transcription through histone lactylation and regulates protein functions via non-histone lactylation, thereby promoting tumorigenesis across multiple cancer types [2–6]. However, the underlying mechanisms by which lactic acid suppresses antitumor immunity remain complex and not fully elucidated.

Endothelial cell-specific molecule 1 (ESM1), a multifunctional secreted protein, is implicated in tumor proliferation, drug resistance, angiogenesis, and immune evasion [7–9]. Our previous work demonstrated that ESM1 induces vasculogenic mimicry and angiogenesis through metabolic reprogramming [3]. Notably, lactate enhances ESM1 expression in ovarian cancer, which suppresses CD8+ T-cell-mediated immune cytotoxicity and induces cisplatin resistance via the SCD1/Wnt signaling axis [10]. Cisplatin, a chemotherapeutic agent, triggers DNA damage response (DDR) in tumor cells, generating cytosolic double-stranded DNA (dsDNA), a key signal for CD8+ T-cell infiltration [11–13]. However, whether ESM1 reduces dsDNA production by diminishing tumor cell sensitivity to cisplatin, thereby reducing immune-mediated tumor elimination, remains unknown.

Independent studies, including ours, have identified the Akt1 pathway as a critical downstream signaling axis of ESM1 in tumor progression [3,7,14]. MDM2, an oncogenic effector of Akt1, promotes ubiquitination-mediated degradation of p53, attenuating DDR [15]. In tumor cells, DDR-induced cytosolic dsDNA accumulation activates the cGAS-STING pathway: cGAS homodimerizes and binds dsDNA in a sequence-independent manner, transitioning from an autoinhibited state to an active conformation that synthesizes the second messenger cGAMP [16]. cGAMP binds STING, triggering its translocation from the endoplasmic reticulum to the Golgi apparatus, where it recruits TBK1/IKKε to phosphorylate IRF3 [17]. Phosphorylated IRF3 dimerizes and translocates to the nucleus, driving interferon and pro-inflammatory cytokine production to enhance antitumor immunity [18]. Despite these insights, whether ESM1 suppresses the cGAS-STING pathway via Akt1 activation to impair tumor immunity has not been reported.

To elucidate the molecular mechanisms by which lactate promotes tumor DDR and subsequent immune evasion, we conducted a systematic investigation through a series of comprehensive experiments.

## 2 Methods

### 2.1 Bioinformatics Analysis

Transcriptomic profiles of HCC, OC, and LUAD were extracted from the TCGA database (https://portal.gdc.cancer.gov/). ESM1 expression levels and immune cell infiltration were analyzed using the ssGSEA (single-sample Gene Set Enrichment Analysis), CIBERSORT (Cell-type Identification By Estimating Relative Subsets of RNA Transcripts), and ESTIMATE (Estimation of STromal and Immune cells in MAlignant Tumors using Expression data) algorithms, with specific methodologies referenced from our previously published protocols [7].

### 2.2 Clinical Patient Samples

In the Department of Pathology at Zhuzhou Hospital Affiliated to Xiangya School of Medicine, we collected 50 OC patient samples, 43 LUAD patient samples, and 34 HCC patient samples. This study involving human clinical samples was conducted in accordance with the ethical principles of the Declaration of Helsinkiand approved by the Ethics Committee of Zhuzhou Hospital Affiliated to Xiangya School of Medicine (Approval No.: #2025-KY-127). Written informed consent was obtained from all participants prior to sample collection. For anonymized data derived from clinical samples, participant identities were encrypted and stored securely to comply with data protection regulations. Any identifying information in the manuscript has been removed or anonymized to ensure privacy.

### 2.3 Cell Culture

The human tumor cell lines (CAOV3, A549, Hep3B) and murine-derived cancer cell lines (ID8, Hepa1-6, LLC) were purchased from the American Type Culture Collection (ATCC, Manassas, VA, USA). CAOV3, A549, ID8, Hepa1-6, and LLC cells were cultured in DMEM medium (Shanghai Zhong Qiao Xin Zhou Biotechnology Co., Ltd., ZQ-100, Shanghai, China), while Hep3B cells were maintained in EMEM medium (Shanghai Zhong Qiao Xin Zhou Biotechnology Co., Ltd., ZQ-302, China). All cells have been confirmed to be correct through STR identification, and there is no obvious mycoplasma infection. STR identification data for the cell lines are available from the authors upon reasonable request. All media were supplemented with 10% fetal bovine serum (FBS; Gibco, Invitrogen, Carlsbad, CA, USA) and 1% penicillin-streptomycin (P/S; GIBCO, Carlsbad, CA, USA). Cells were incubated in a humidified atmosphere at 37°C with 5% CO_2_. The ESM1 short hairpin RNA (shRNA) (The target sequence information for ESM1sh#1 is GCATCTGGAGATGGCAATATT, and the target sequence information for ESM1sh#2 is GCAATAATTATGCGGTGGACT.), ESM1 overexpression (OE) plasmid, lactate, Akt1 inhibitor, and corresponding vector controls were obtained from HonorGene Biotechnology Co., Ltd. (Changsha, China). Transfection was performed using Lipofectamine®3000 (Thermo Fisher Scientific, Inc., Waltham, MA, USA) following the manufacturer’s protocol. *In vitro* experiments, the Akt1 inhibitor LY294002 was administered at concentrations of 20 μM for A549 cells, 10 μM for Hep3B cells, and 10 μM for CAOV3 cells.

### 2.4 Cell Function Experiment

EdU staining and flow cytometry, please refer to our previous studies [7,10]. For comet assay, the cancer cells were seeded into 6-well plates and incubated overnight at 37°C with 5% CO_2_. DNA damage was measured using the Comet Assay Kit (KeyGEN BioTECH, Nanjing, China) according to the manufacturer’s protocol for single-cell gel electrophoresis. Briefly, cells were resuspended in ice-cold PBS at a density of 1 × 10^6^ cells/mL. Agarose gels of varying concentrations were prepared, followed by cell lysis. The slides were maintained horizontally and carefully transferred from the alkaline solution to a horizontal electrophoresis chamber. Electrophoresis was then performed in an alkaline buffer. Subsequently, the slides were stained with propidium iodide (PI) and visualized under a fluorescence microscope. DNA damage quantification employed CASP software (v1.2.2) to analyze the following core parameters: Tail Length (μm), Tail Moment (μm × %DNA), and Olive Tail Moment (%DNA × distance^2^), where the Olive Tail Moment reflects the heterogeneous distribution of damaged DNA through weighted calculation.

### 2.5 PCR Analysis

The treated cells were cultured to an appropriate density, subsequently washed with PBS buffer, and then 1 mL of TRIzol (Vazyme, R401-01, Vazyme Biotech Co., Ltd., Nanjing, China) was added to each treatment group for total RNA extraction according to the manufacturer’s instructions. Following RNA isolation, cDNA was synthesized using a reverse transcription kit (Vazyme, R223, Vazyme Biotech Co., Ltd., Nanjing, China) in accordance with the experimental protocol. After obtaining cDNA, a mixture containing cDNA, ddH_2_O, target gene-specific primers, and 2× Rapid Taq Master Mix (Vazyme, P222, Vazyme Biotech Co., Ltd., Nanjing, China) was prepared following the manufacturer’s guidelines of the rapid PCR reagents. The PCR reaction was performed using an Eppendorf Mastercycler (RX40) (Eppendorf, Hamburg, Germany). The resulting products were then analyzed via 2% agarose gel electrophoresis. The primer sequence is shown below: IFN-β, F: 5^′^-ACGCCGCATTGACCATCTAT-3^′^; R: 3^′^-TGGCCTTCAGGTAATGCAGA-5^′^. CCL2, F: 5^′^-CCTAGCTTTCCCCAGACACC-3^′^; R: 3^′^-AAAAGCAATTTCCCCAAGTCTC-5^′^. CCL5, F: 5^′^-CCTCGCTGTCATCCTCATTGCT-3^′^; R: 3^′^-ATCCTTGACCTGTGGACGACT-5^′^. CXCL10, F: 5^′^-CTGCAAGCCAATTTTGTCCAC-3^′^; R: 3^′^-CAGTAAATTCTTGATGGCCTTCG-5^′^. β-actin, F: 5^′^-ACCCTGAAGTACCCCATCGAG-3^′^; R: 3^′^-AGCACAGCCTGGATAGCAAC-5^′^.

### 2.6 Western Blot

The cells were lysed using a cold lysis buffer. The proteins obtained from the lysate were separated using a 10% Sodium Dodecyl Sulfate—Polyacrylamide Gel Electrophoresis (SDS-PAGE) gel, followed by electrophoresis for 60–90 min. The resolved proteins were then transferred onto a Polyvinylidene difluoride (PVDF) membrane. Subsequently, the membrane was blocked with 5% skimmed milk under continuous agitation for 2 h. Next, the membrane was incubated with the primary antibody at 4°C overnight. Afterward, the membrane was exposed to a secondary antibody (goat anti-rabbit/mouse IgG conjugated with Horseradish Peroxidase (HRP; CWBIO, Cat. No. CW0103S, diluted 1:2000) for 2 h at room temperature. Finally, the resulting bands were visualized using an ECL detection reagent. The primary antibodies used were as follows: ESM1 (Abcam, ab224591, diluted 1:1000), Akt1 (Abcam, ab8805, diluted 1:500), p-Akt1-T308 (Abcam, ab38449, diluted 1:1000), MDM2 (Abcam, ab16895, diluted 1:1000), p-MDM2-S166 (Abcam, ab170880, diluted 1: 5000), p53 (Abcam, ab131442, diluted 1:5000), cGAS (Abcam, ab302617, diluted 1:1000), STING (Abcam, ab239074, diluted 1:1000), p-STING (Abcam, ab318181, diluted 1:1000), IRF3 (Abcam, ab68481, diluted 1:1000), p-IRF3-S396 (Abcam, ab320083, diluted 1:1000), β-actin (Abcam, ab8226, diluted 1:10000).

### 2.7 IHC Staining

The immunohistochemical procedure using the two-step detection kit (ZSBG-BIO, PV9000, Beijing, China) was performed as follows: First, tissue sections were sequentially immersed in three fresh xylene baths for 20 min each to remove paraffin. After removing excess liquid, the sections were dehydrated through a graded ethanol series (absolute ethanol, 95% ethanol, and 75% ethanol) for 6 min each. Following a 1-min rinse with distilled water, the sections were equilibrated in PBS buffer. Antigen retrieval was conducted by high-pressure heat-mediated treatment in citrate-based repair solution (pH 6.0) for 15 min. After cooling to room temperature, endogenous peroxidase activity was blocked using a specific blocking agent at 37°C for 20 min, followed by three PBS washes. The sections were then incubated with the primary antibody at 37°C for 2 h and washed three times with PBS. An enhancer reagent was applied and incubated at 37°C for 20 min to amplify the signal, followed by another three PBS washes. Subsequently, the tissue was incubated with secondary antibody at room temperature for 20 min and washed three times with PBS. Visualization was achieved using the 3,3^′^-diaminobenzidine (DAB) chromogenic kit (ZSBG-BIO, ZLI-9018, Beijing, China) under microscopic monitoring. Finally, the sections were counterstained with hematoxylin, dehydrated through a graded ethanol series, and mounted for microscopic analysis. The primary antibodies used are shown below: PCNA (Abcam, Cambridge, UK, ab29, diluted 1:500), ESM1 (Abcam, Cambridge, UK, ab224591, diluted 1:300), dsDNA (Abcam, ab273137, diluted 1:500) and CD8 (Abcam, Cambridge, UK, ab245118, diluted 1:500). The criteria for IHC staining score can be referred to in our previous study [19]. The immunohistochemical evaluation employed a dual-parameter scoring system to quantify protein expression levels. Staining intensity was categorized into three tiers: absent (0), weak (1), and intense (2) chromogenic reactions. Concurrently, the extent of positive cellular infiltration was graded by quantitative assessment: <5% (0), 5%–25% (1), 26%–50% (2), and >50% (3) of cells exhibiting specific immunoreactivity. The composite score derived from the arithmetic summation of intensity and distribution parameters enabled classification into distinct expression phenotypes: composite scores ≥2 indicated high expression patterns, whereas values <2 were designated as low-expression status. Finally, Spearman’s rank correlation analysis was performed to evaluate the association between variables based on IHC scoring.

### 2.8 IF Staining

Cells were fixed with 4% paraformaldehyde (AWI0056B, Abiowell, Changsha, China) for 30 min at room temperature, followed by permeabilization with 0.5% Triton X-100. Nonspecific binding was blocked using 5% bovine serum albumin (BSA), and primary antibodies were applied to the cells at 4°C overnight (12 h). After washing, samples were incubated with fluorescence-conjugated secondary antibodies (AWS0003; Abiowell, Changsha, China) for 2 h under ambient conditions. Nuclei were counterstained with DAPI (1 μg/mL) for 5 min, and sections were imaged using a fluorescence microscope.

### 2.9 TUNEL Staining

CAOV3, Hep3B, and A549 cells were seeded into 6-well plates at a density of 2 × 10^5^ cells/well and cultured overnight to reach ~70% confluence. After discarding the culture medium, cells were fixed with 4% paraformaldehyde for 30 min at room temperature, followed by permeabilization with 0.1% Triton X-100 in PBS for 2 min on ice. TUNEL staining was performed using a commercial apoptosis detection kit (Wanleibio, # WLA127, Shenyang, China) according to the manufacturer’s instructions: 50 μL of TUNEL reaction mixture (containing terminal deoxynucleotidyl transferase and FITC-dUTP) was added to each well and incubated for 1 h at 37°C in a humidified dark chamber. Negative controls omitted the TdT enzyme, while positive controls were pre-treated with DNase I (3 U/mL) for 10 min. Post-reaction washing (3 × 5 min PBS) was followed by DAPI nuclear counterstaining (5 μg/mL, 5 min). Fluorescent signals were visualized using a confocal microscope, and apoptosis rates were quantified by counting TUNEL-positive nuclei relative to total DAPI-stained nuclei in ≥5 random fields per sample.

### 2.10 Xenograft Tumor Model

The Esm1 knock-out female mouse model was purchased from Shanghai Model Organisms Center, Inc. (Shanghai, China). In brief, the establishment process of the Esm1 knockout mouse model involved the following steps: (1) Cas9 mRNA and sgRNAs were generated via *in vitro* transcription; (2) a mixture of Cas9 mRNA and sgRNAs was microinjected into fertilized eggs (C57BL/6J strain); (3) F0-generation mice were generated and screened via PCR and sequencing; (4) F0-positive mice were crossed with wild-type C57BL/6J mice to produce F1 offspring. The sequences of sgRNAs were: gRNA1 (ACGGGGTCAAGTGTGGTCCG GGG) and gRNA2 (GTACAGTCTCAGGCATGGAC GGG).

Murine-derived cancer cell lines (ID8, Hepa1-6, or LLC) were subcutaneously injected into 6-week-old Esm1 knock-out mice at a density of 5 × 10^6^ cells per animal (Three in each group). Tumor growth was monitored weekly, and average tumor volume was calculated from day 7 until humane endpoint euthanasia on day 36, with three biological replicates per group. Following resection, xenograft tumors were weighed and subsequently fixed in formalin, paraffin-embedded, and subjected to immunohistochemical (IHC) staining, ELISA, or flow cytometry. Humane endpoints: Euthanasia performed when tumor diameter exceeded 15 mm or body weight loss ≥20%.

Animal welfare monitoring: Daily checks for morbidity/mortality by trained staff; veterinary care provided as needed. All experimental procedures align with ARRIVE Essential 10 and the 3Rs principles (Replacement, Reduction, Refinement).

All animal experiments were performed following the Guide for the Care and Use of Laboratory Animals (NIH, USA) and approved by the Institutional Animal Care and Use Committee (IACUC) of Zhuzhou Hospital Affiliated to Xiangya School of Medicine (Approval No.: #20240968-2). The study adhered to the “3R principles” (Replacement, Reduction, Refinement) to minimize animal suffering. Surgical procedures were conducted under anesthesia, and humane endpoints were strictly implemented.

### 2.11 Statistical Analyses

Statistical analyses were conducted using GraphPad Prism 8.0 (GraphPad, Inc., USA). One-way ANOVA was utilized for comparisons within each group, while an independent samples *t*-test was applied for comparisons between two groups. For comparisons among multiple groups, one-way ANOVA was performed following a variance homogeneity test. Data processing and hypothesis testing were implemented in the R programming language (version 4.3.2). Each experiment was repeated at least three times, and the data are presented as means ± SD. Statistical significance was defined as *p*-values < 0.05. *p < 0.05, **p < 0.01, ***p < 0.001. NS, not significant.

## 3 Results

### 3.1 Lactate Upregulate ESM1 mRNA and Protein Expression in Cancer Cells Exposed to Cisplatin

**Figure 1:**
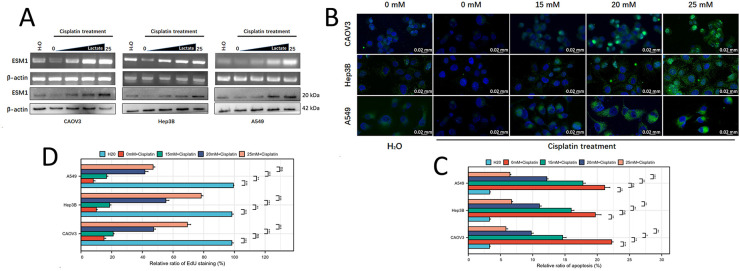

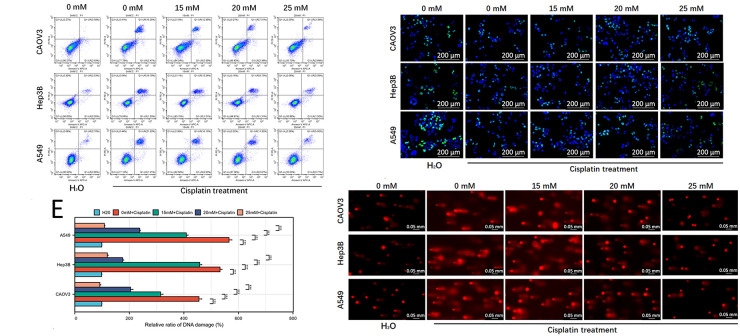
The lactate concentration gradient promotes ESM1 expression in tumor cells and suppresses DNA damage. (**A**). PCR and WB to examine the effects of lactate concentration gradients on ESM1 expression in OC, HCC, and LUAD cells. (**B**). IF staining to validate the effects of lactate concentration gradients on ESM1 expression in OC, HCC, and LUAD cells. scale bar: 20 μm. (**C**). EdU assay to detect the effects of lactate concentration gradients on the proliferative capacity of OC, HCC, and LUAD cells under cisplatin exposure. scale bar: 200 μm. ns *p* > 0.05, *p < 0.05, **p < 0.01, ***p < 0.001. (**D**). Flow cytometry to assess the effects of lactate concentration gradients on the apoptotic capacity of OC, HCC, and LUAD cells under cisplatin exposure. ns *p* > 0.05, **p < 0.01, ***p < 0.001. (**E**). Comet assay to evaluate the effects of lactate concentration gradients on DNA damage levels in OC, HCC, and LUAD cells under cisplatin exposure. scale bar: 50 μm. ***p < 0.001. ESM1: Endothelial Cell-Specific Molecule 1; PCR: Polymerase Chain Reaction; WB: Western Blot; OC: Ovarian Cancer; HCC: Hepatocellular Carcinoma; LUAD: Lung Adenocarcinoma; EdU: 5-ethynyl-2^′^-deoxyuridine.

In our previous studies, we demonstrated that lactate promotes both transcriptional and translational upregulation of ESM1 [10]. To further elucidate the multifaceted role of lactate in tumor DDR, we treated ovarian cancer (OC), hepatocellular carcinoma (HCC), and lung adenocarcinoma (LUAD) cells with cisplatin to induce DNA damage and exposed them to gradient concentrations of lactate. PCR and Western blot analyses revealed that lactate dose-dependently increased ESM1 mRNA and protein expression levels in cisplatin-treated OC, HCC, and LUAD cells (Figs. 1A and S1). Immunofluorescence staining further corroborated these findings, showing enhanced ESM1 expression in a lactate concentration-dependent manner (Fig. 1B).

### 3.2 Lactate Promotes Cancer Cell Proliferation and Suppresses Apoptosis and DNA Damage under Cisplatin Exposure

Subsequently, we further investigated the effect of lactate on cisplatin-induced anti-proliferative activity in cancer cells via EdU staining assays. The results revealed that lactate counteracted the tumor-killing effect of cisplatin in a concentration-dependent manner (Fig. 1C). Flow cytometry apoptosis assays demonstrated that lactate restored cisplatin-mediated tumor cell apoptosis in a concentration-gradient-dependent fashion (Fig. 1D). Further comet assays indicated that lactate attenuated cisplatin-induced DNA damage in cancer cells, also in a concentration-dependent manner (Fig. 1E). These results suggest that lactate may serve as a key metabolite antagonizing DNA damage in tumor cells.

### 3.3 ESM1 Serves as a Critical Downstream Protein Mediating Lactate-Induced Antagonism of Tumor DNA Damage

**Figure 2:**
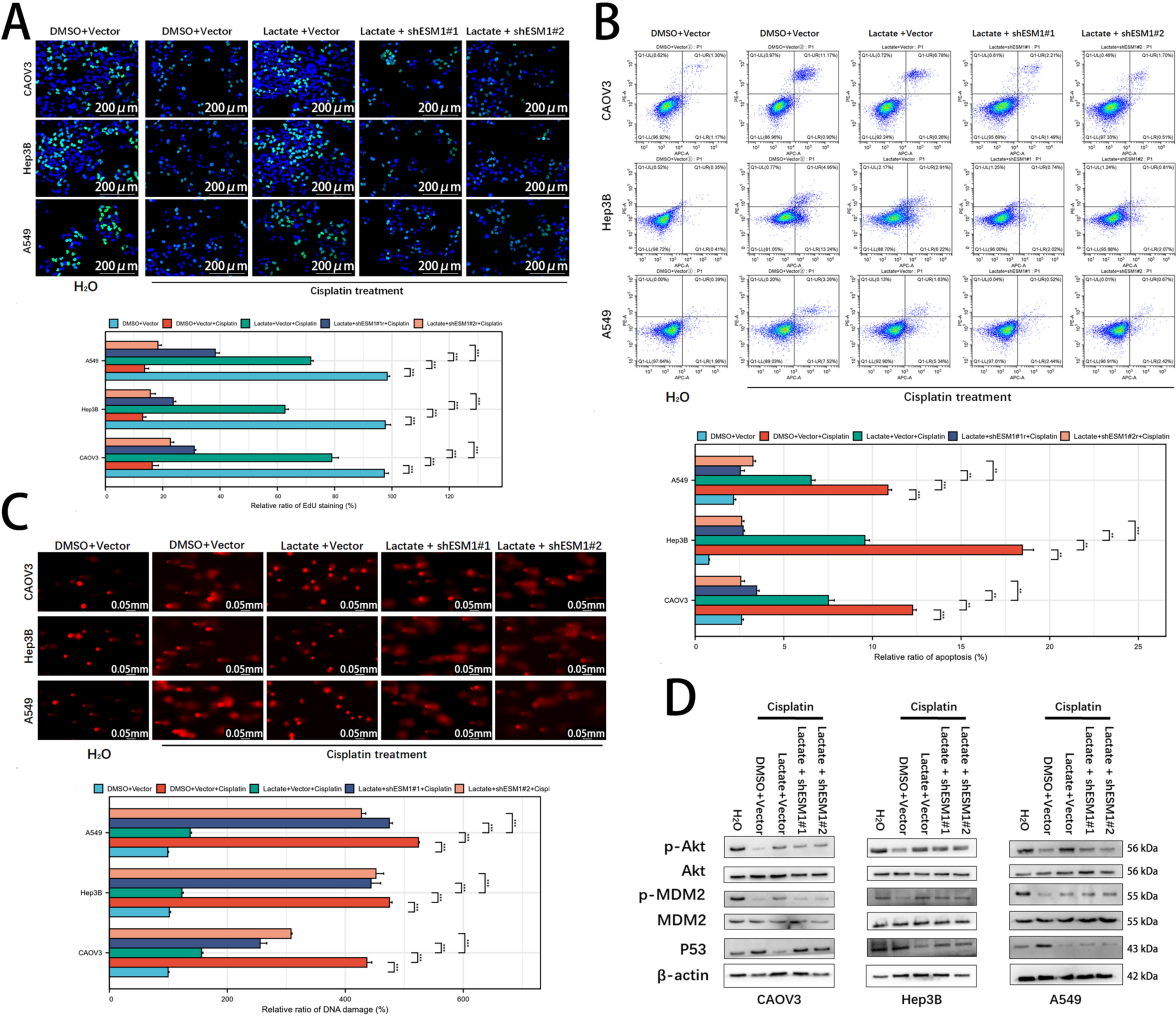
Lactate suppresses tumor DDR and activates the Akt1-MDM2-p53 signaling pathway via ESM1. (**A**). EdU assay to examine the effects of the lactate-ESM1 signaling axis on the proliferative capacity of OC, HCC, and LUAD cells under cisplatin exposure. scale bar: 200 μm. ***p < 0.001. (**B**). Flow cytometry-based apoptosis assay to assess the effects of the lactate-ESM1 signaling axis on the apoptotic capacity of OC, HCC, and LUAD cells under cisplatin exposure. **p < 0.01, ***p < 0.001. (**C**). Comet assay to evaluate the effects of the lactate-ESM1 signaling axis on DNA damage levels in OC, HCC, and LUAD cells under cisplatin exposure. scale bar: 50 μm. ***p < 0.001. (**D**). WB to detect the effects of the lactate-ESM1 signaling axis on the expression levels of p-Akt1, Akt1, p-MDM2, MDM2, and p53 proteins. DDR: DNA Damage Repair; Akt1: Protein Kinase B (AKT) Serine/Threonine Kinase 1; MDM2: Murine Double Minute 2; ESM1: Endothelial Cell-Specific Molecule 1; OC: Ovarian Cancer; HCC: Hepatocellular Carcinoma; LUAD: Lung Adenocarcinoma; WB: Western Blot; DMSO: Dimethyl Sulfoxide.

In our previous study, we demonstrated that lactate upregulates ESM1 expression in ovarian cancer cells and antagonizes cisplatin-induced cytotoxicity [10]. EdU staining assays revealed that ESM1 knockdown under cisplatin exposure significantly attenuated the lactate-mediated pro-proliferative effect (Fig. 2A). Flow cytometry apoptosis assays further confirmed that ESM1 silencing weakened the anti-apoptotic capacity of lactate (Fig. 2B). Comet assays indicated that ESM1 knockdown partially reversed the lactate-induced suppression of DNA damage (Fig. 2C). Mechanistically, Akt1 was identified as a downstream signaling pathway of ESM1 [7]. Given that MDM2, a key Akt1 effector, facilitates DNA repair by suppressing p53 [20–22]. We examined the regulatory role of the lactate-ESM1 axis on the Akt1-MDM2-p53 pathway. Western blot analysis demonstrated that lactate activates the Akt1-MDM2 signaling cascade via ESM1, thereby reducing p53 expression levels in cisplatin-treated tumor cells (Figs. 2D and S2). These findings collectively suggest that ESM1 serves as a critical mediator of lactate-induced antagonism against DNA damage in tumor cells, a process tightly associated with Akt1-MDM2 pathway activation.

### 3.4 ESM1 Inhibits cGAS-STING Signaling through Akt1 Activation to Attenuate DNA Damage in Tumor Cells

**Figure 3:**
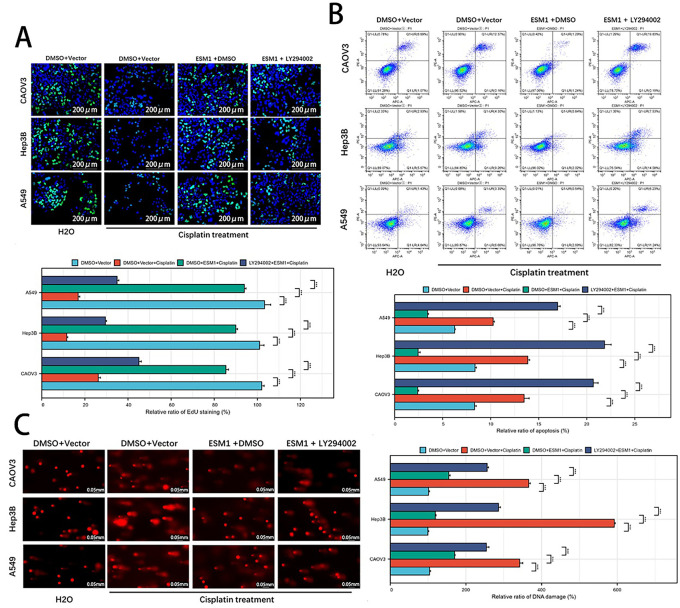

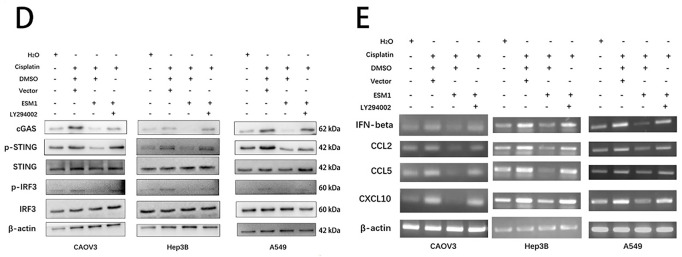
ESM1 suppresses tumor cell DNA damage and inhibits cGAS-STING pathway activation via the Akt1 signaling pathway. (**A**). EdU assay to examine the effects of the ESM1-Akt1 signaling axis on the proliferative capacity of OC, HCC, and LUAD cells under cisplatin exposure. scale bar: 200 μm. ***p < 0.001. (**B**). Flow cytometry-based apoptosis assay to assess the effects of the ESM1-Akt1 signaling axis on the apoptotic capacity of OC, HCC, and LUAD cells under cisplatin exposure. ***p < 0.001. (**C**). Comet assay to evaluate the effects of the ESM1-Akt1 signaling axis on DNA damage levels in OC, HCC, and LUAD cells under cisplatin exposure. scale bar: 50 μm. ***p < 0.001. (**D**). WB to detect the effects of the ESM1-Akt1 signaling axis on the expression of cGAS, p-STING, STING, p-IRF3, and IRF3 proteins in OC, HCC, and LUAD cells under cisplatin exposure. (**E**). PCR to analyze the effects of the ESM1-Akt1 signaling axis on the transcription of IFN-β, CCL2, CCL5, and CXCL10 in OC, HCC, and LUAD cells under cisplatin exposure. In these experiments, the dosage treatments of LY294002 were 20 μM for A549 cells, 10 μM for Hep3B cells, and 10 μM for CAOV3 cells. EMS1: Endothelial Cell-Specific Molecule 1; cGAS: Cyclic GMP-AMP Synthase; STING: Stimulator of Interferon Genes; Akt1: Protein Kinase B (AKT) Serine/Threonine Kinase 1; EdU: 5-ethynyl-2^′^-deoxyuridine; OC: Ovarian Cancer; HCC: Hepatocellular Carcinoma; LUAD: Lung Adenocarcinoma; WB: Western Blot; IRF3: Interferon Regulatory Factor 3; IFN-β: Interferon Beta; CCL2: Chemokine (C–C Motif) Ligand 2; CCL5: Chemokine (C–C Motif) Ligand 5; CXCL10: Chemokine (C–X–C Motif) Ligand 10.

To investigate whether ESM1 inhibits tumor cell proliferation, apoptosis, and DDR through the Akt1 pathway, experimental results demonstrated the following: EdU assays revealed that ESM1 reversed cisplatin-induced suppression of proliferation, while the Akt1 inhibitor LY294002 counteracted the pro-proliferative effects of ESM1 overexpression (Fig. 3A). Flow cytometry apoptosis analysis showed that ESM1 attenuated cisplatin-promoted apoptosis, and LY294002 antagonized the anti-apoptotic effects of ESM1 overexpression (Fig. 3B). Comet assays further indicated that cisplatin-induced DDR was mitigated by ESM1 overexpression, and this protective effect was abolished by Akt1 inhibition (Fig. 3C), suggesting ESM1 reduces DDR via Akt1 signaling. Since DNA damage triggers dsDNA accumulation and activates the cGAS-STING pathway [18], WB demonstrated that ESM1 suppressed the expression of cGAS, phosphorylated STING (p-STING), and phosphorylated IRF3 (p-IRF3) (Fig. 3D). PCR experiments confirmed that ESM1 inhibited the transcription of key cGAS-STING downstream cytokines, including IFN-β, CCL2, CCL5, and CXCL10, with LY294002 reversing these effects (Fig. 3E). Since Akt includes three isotypes and LY294002 exerts inhibitory effects on all three Akt isotypes, we employed Akt1-specific inhibitor Akt1-IN-1 to perform rescue experiments targeting the ESM1-Akt1 signaling axis. The results demonstrated that Akt1-IN-1 significantly antagonized the pro-proliferative, anti-apoptotic, and DDR capacities induced by ESM1 overexpression (Fig. S3A–C). These findings collectively indicate that ESM1 suppresses the cGAS-STING pathway through Akt1 signaling, thereby reducing DNA damage in cancer cells.

### 3.5 ESM1 Inhibit CD8+ T Cell Infiltration to Promote Cancer Growth In Vivo

**Figure 4:**
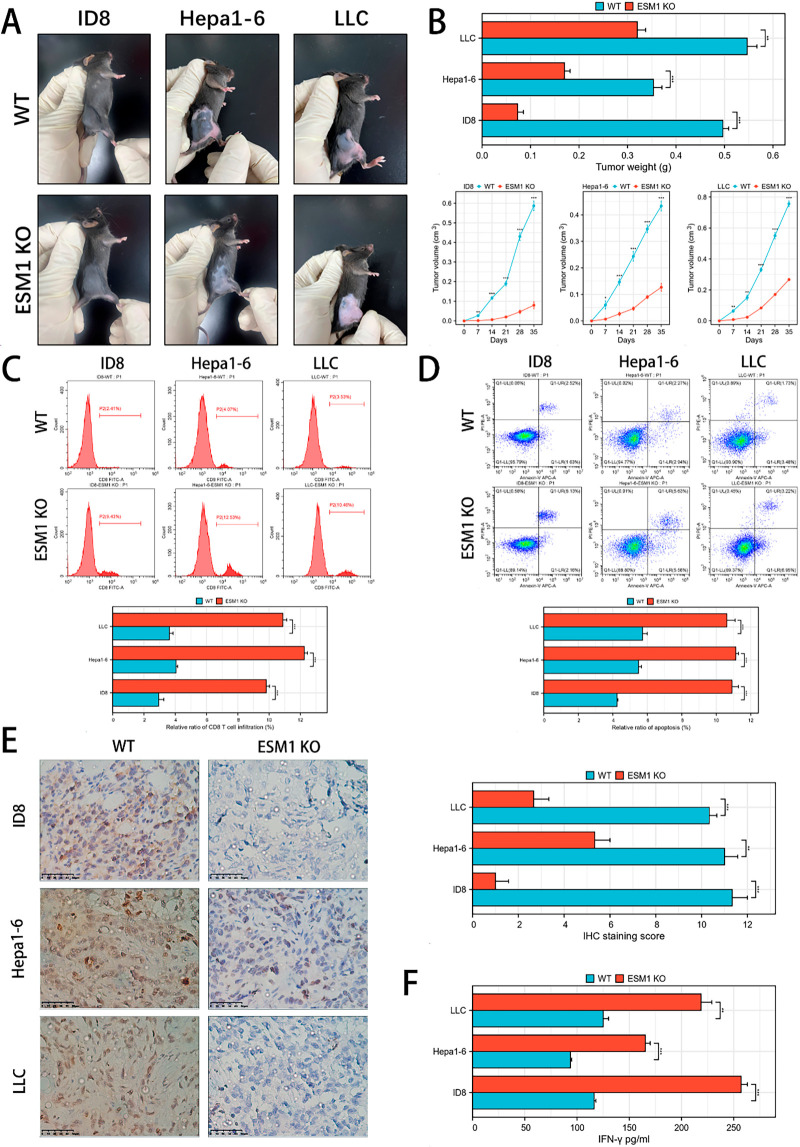
*In vivo* experiments validate that ESM1 inhibits CD8+ T cell infiltration to promote tumor apoptosis. (**A**). Establish a xenograft tumor model using ESM1 whole-gene knockout mice to examine the effects of ESM1 knockout on tumor cell growth *in vivo*. (**B**). Analyze tumor volume and weight in each group. *p < 0.05 **p < 0.01, ***p < 0.001. (**C**). Flow cytometry to detect CD8+ T cell infiltration in the xenograft tumors. ***p < 0.001. (**D**). Flow cytometry-based apoptosis assay to assess apoptotic levels in the xenograft tumors. ***p < 0.001. (**E**). IHC staining to evaluate the effects of ESM1 knockout on PCNA expression in the xenograft tumors. scale bar: 50 μm. **p < 0.01, ***p < 0.001. (**F**). ELISA to determine the impact of ESM1 on IFN-γ expression in the xenograft tumors. **p < 0.01, ***p < 0.001. ESM1: Endothelial Cell-Specific Molecule 1; CD8+: Cluster of Differentiation 8 Positive; IHC: Immunohistochemistry: PCNA: Proliferating Cell Nuclear Antigen; ELISA: Enzyme-Linked Immunosorbent Assa; IFN-γ: Interferon Gamma; WT: Wild Type; ESM1 KO: Endothelial Cell-Specific Molecule 1 Knockout.

To impact of ESM1 on CD8+ T cell infiltration in the tumor microenvironment (TME) *in vivo*, we generated ESM1 knockout (KO) mice and established xenograft models of hepatocellular carcinoma, lung cancer, and ovarian cancer. Compared to wild-type (WT) mice, ESM1 KO mice exhibited significantly reduced tumor volume and weight (Fig. 4A,B). Flow cytometry analysis revealed a marked increase in CD8+ T cell infiltration within the tumors of KO mice (Fig. 4C), accompanied by elevated apoptosis levels (Fig. 4D). IHC staining demonstrated decreased PCNA expression in ESM1 KO tumors (Fig. 4E), indicating suppressed tumor proliferation. Additionally, ELISA assays showed higher IFN-γ levels in the KO group (Fig. 4F). Furthermore, we also employed HE staining to detect morphological differences in multiple organs between the two groups of mice to determine whether ESM1-KO mice exhibited congenital developmental disparities compared to wild-type mice. The results showed no significant differences in the heart, liver, spleen, lung, or kidney morphology between ESM1-KO mice and wild-type mice. (Fig. S4). These findings collectively suggest that removal of ESM1 in the TME enhances CD8+ T cell infiltration, promotes tumor cell apoptosis, and inhibits tumor growth, likely through mechanisms involving IFN-γ-mediated immune activation and altered T cell dynamics. The observed correlation between reduced ESM1 and enhanced antitumor immunity aligns with studies highlighting the regulatory role of microenvironmental factors in CD8+ T cell exhaustion and function.

### 3.6 Clinical Validation of ESM1 Expression and CD8+ T Cell Infiltration in Cancer Patient Samples

**Figure 5:**
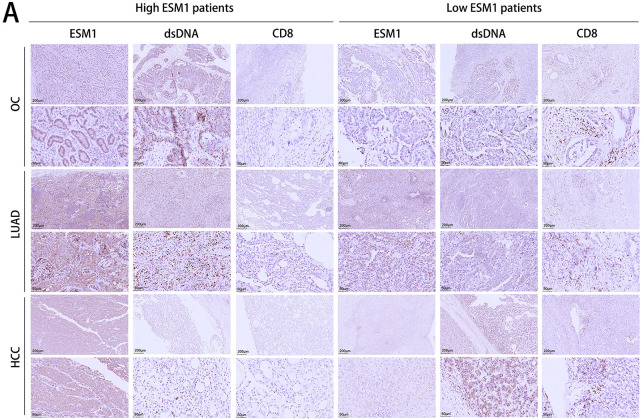

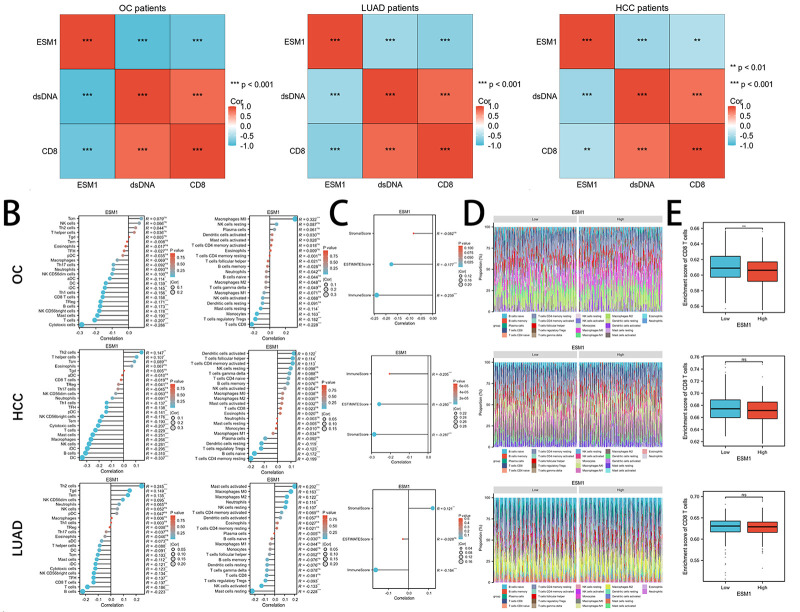
Detect the expression levels and correlation between ESM1 and CD8+ T cells in patient samples of OC, HCC, and LUAD. (**A**). IHC was used to detect the expression levels of ESM1, dsDNA, and CD8 in OC, LUAD, and HCC patient samples, followed by correlation analysis. scale bar: 50 or 200 μm. **p < 0.01, ***p < 0.001. (**B**). Based on the TCGA database, the CIBERSORT algorithm was applied to analyze the correlation between ESM1 expression and various immune cell types in patients with OC, LUAD, and HCC. ns *p* > 0.05, *p < 0.05, **p < 0.01, ***p < 0.001. (**C**). The ESTIMATE algorithm was used to analyze the correlation between ESM1 expression and stromal/immune scores. ns *p* > 0.05, **p < 0.01, ***p < 0.001. (**D**). Based on the CIBERSORT algorithm, 22 immune cell markers were analyzed to characterize immune infiltration in the three TCGA datasets. (**E**). Bioinformatics analysis was performed to analyze CD8+ T cell infiltration levels in tumor samples with high and low ESM1 expression based on the TCGA database. ns *p* > 0.05, **p < 0.01. ESM1: Endothelial Cell-Specific Molecule 1; CD8+: Cluster of Differentiation 8 Positive; OC: Ovarian Cancer; HCC: Hepatocellular Carcinoma; LUAD: Lung Adenocarcinoma; TCGA: The Cancer Genome Atlas; CIBERSORT: Cell-Type Identification by Estimating Relative Subsets of RNA Transcripts; ESTIMATE: Estimation of STromal and Immune Cells in MAlignant Tumour Tissues Using Expression Data.

To further confirm whether ESM1-induced CD8+ T cell infiltration through DDR genuinely exists in cancer patients, we analyzed tumor patient samples to detect the expression of ESM1, dsDNA, and CD8. The results revealed that in patients with high ESM1 expression, dsDNA and CD8 levels were significantly reduced. Conversely, in tumors with low ESM1 expression, dsDNA and CD8 expression were markedly higher (Fig. 5A). Further correlation analysis demonstrated a significantly negative correlation between ESM1 and both dsDNA and CD8 expression, whereas dsDNA and CD8 expression showed a positive correlation (Fig. 5A). Bioinformatics serves as a cornerstone in advancing contemporary molecular medicine research [23,24]. Therefore, using the TCGA database, we investigated the immune infiltration patterns associated with ESM1 in HCC, OC, and LUAD. The ssGSEA algorithm indicated a significant negative correlation between ESM1 and T cell infiltration across all three cancer types. The CIBERSORT algorithm revealed that ESM1 was significantly negatively correlated with CD8+ T cell infiltration in ovarian cancer patients, but no significant differences were observed in HCC or LUAD. The ESTIMATE algorithm further demonstrated that ESM1 expression was negatively correlated with Immune Scores in all three cancer types. Moreover, patients stratified by high or low ESM1 expression exhibited significant differences in immune cell infiltration abundance. Specifically, ovarian cancer patients with low ESM1 expression displayed higher CD8+ T cell infiltration compared to high-ESM1 expressers. Although CD8+ T cell infiltration levels in HCC and LUAD patients did not reach statistical significance between high- and low-ESM1 groups, the average infiltration in low-ESM1 patients remained higher than in high-ESM1 cohorts (Fig. 5B–E).

## 4 Discussion

Lactic acid, as a critical byproduct in tumor metabolism, has been demonstrated to play significant roles in tumor DDR and immune infiltration [25–28]. However, there remains insufficient research to definitively establish whether lactic acid can influence tumor immune infiltration through DDR. In our study, we found that lactate can inhibit DNA damage in OC, LUAD and HCC cells in a concentration-dependent manner (10, 15, 20, and 25 mM). This is consistent with findings from multiple laboratories, such as the study by Zheng et al. demonstrating that malic enzyme 2-derived lactate facilitates the development of acquired chemoresistance in cancer cells undergoing prolonged chemotherapy, primarily through promoting lactylation of proteins involved in homologous recombination repair [29]. Lu et al. discovered that lactate induces histone H4K12 lactylation(H4K12la) through lactate accumulation, which upregulates super enhancer-mediated aberrant RAD23A expression, thereby enhancing DNA damage repair capacity and promoting chemoresistance in OC cells [30]. Wagner et al. discovered that lactate-treated cells exhibited nearly a 2-fold increase in the expression of LIG4, NBS1, and APTX, along with enhanced DNA-PKcs activity, thereby promoting DNA damage repair processes and strengthening cervical cancer cell resistance to anticancer therapies [31]. Therefore, these findings indicate that lactate serves as a critical metabolic regulator suppressing the DDR in tumor cells.

ESM1, a critical tumor-associated secretory protein, has been demonstrated in our previous studies to exert significant pro-tumorigenic roles in OC [19] and LUAD [14]. Additionally, as a key downstream gene of lactate within the tumor microenvironment, ESM1 suppresses CD8+ T cell infiltration in OC and reduces the anticancer efficacy of cisplatin [10]. In this study, we found that ESM1 significantly inhibits DDR in HCC, OC, and LUAD cells, thereby antagonizing the cytotoxic effects of cisplatin on cancer cells. Choi et al. also demonstrated in breast cancer research that benzimidazole derivatives (e.g., mebendazole) can kill breast cancer cells by inducing DNA damage and cell cycle arrest through downregulation of CD44, OCT3/4, and ESM1 [32]. However, no studies to date have elucidated the mechanisms by which ESM1 suppresses DNA damage in tumor cells. In our previous research, we demonstrated that Akt1 can be activated by ESM1 overexpression, with similar effects observed in both KIRC, LUAD, and OC cells [7,14]. The Akt1 pathway suppresses DNA damage through the downstream MDM2-p53 axis, a well-established mechanism in cancer research [33–35]. Numerous studies have demonstrated that lactate accelerates tumor progression via the PI3K-Akt1 pathway [36–38]. Our study further confirms that lactate-ESM1 signaling axis activates the MDM2-p53 pathway through Akt1-mediated mechanisms. Therefore, these results indicate that lactate suppresses the DDR in tumor cells by activating the Akt1-MDM2-p53 pathway through ESM1.

**Figure 6:**
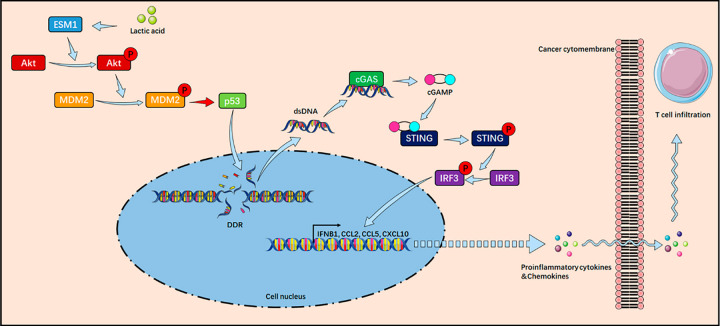
Mechanism diagram illustrating how lactate inhibits the cGAS-STING pathway through the ESM1-Akt1-MDM2-p53 signaling axis to suppress tumor cell DDR and CD8+ T cell infiltration. ESM1: Endothelial Cell-Specific Molecule 1; Akt: Protein Kinase B; MDM2: Murine Double Minute 2; DDR: DNA Damage Repair; dsDNA: Double-Stranded DNA; cGAS: Cyclic GMP-AMP Synthase; cGAMP: Cyclic Guanosine Monophosphate-Adenosine Monophosphate; STING: Stimulator of Interferon Genes; IRF3: Interferon Regulatory Factor 3; IFNB1: Interferon Beta 1; CCL2: Chemokine (C–C Motif) Ligand 2; CCL5: Chemokine (C–C Motif) Ligand 5; CXCL10: C–X–C Motif Chemokine Ligand 10.

In tumor cells, DDR leads to the accumulation of dsDNA, which serves as a critical mediator in inducing antitumor immunity, primarily through the classical activation of the cGAS-STING signaling pathway [17,18,39,40]. In this study, we found that ESM1 inhibits the activation of the cGAS-STING pathway via the Akt1 signaling axis and suppresses the transcription of key downstream chemokines and interferons. *In vivo* experiments further demonstrated that ESM1 knockout significantly enhanced CD8+ T cell infiltration in xenograft tumors and suppressed tumor growth. Researchers led by Ka NL discovered that activation of the cGAS-STING pathway serves as a critical signaling mechanism for inducing anti-cancer immune responses in breast cancer [41]. Chen T et al. also found that restricting intracellular dsDNA accumulation and suppressing cGAS/STING activation promotes tumorigenesis and resistance to anti-PD-L1 immunotherapy [42]. Research by Hong et al. also indicates that dsDNA acts as a critical mediator in activating the cGAS-STING pathway to promote antitumor immunity [43]. Based on OC, HCC, and LUAD patient samples, our IHC staining experiments detecting ESM1, dsDNA, and CD8 expression levels revealed a negative correlation between ESM1 and dsDNA levels, as well as a significant negative correlation between ESM1 and CD8 expression. Conversely, CD8 expression showed a significant positive correlation with dsDNA. These findings further corroborate that ESM1-mediated suppression of CD8+ T cell infiltration is closely associated with dsDNA generated from tumor DNA damage (Fig. 6).

In this study, there are also several limitations. For example, it remains unclear whether ESM1, as a secreted protein, can directly regulate the viability and cytotoxicity of effector T cells after being taken up by them. Additionally, the interactions between ESM1 and other immune cells, including NK cells, regulatory T cells (Tregs), B cells, and dendritic cells (DCs), are still unknown. Furthermore, whether lactate regulates immune cell viability through other molecular mechanisms, or modulates DNA damage repair (DDR) in tumor cells via lactylation or other post-translational modifications, remains to be elucidated.

## 5 Conclusion

Our study demonstrates that the lactate-ESM1 signaling axis attenuates cisplatin-induced tumor cell DNA damage via the Akt1-MDM2-p53 pathway. Furthermore, it inhibits the cGAS-STING signaling pathway to reduce CD8+ T cell infiltration, thereby promoting tumor progression. These findings highlight the pivotal role of the lactate-ESM1 axis in tumor DNA damage response and tumor immunomodulation.

## Supplementary Materials



## Data Availability

The data that support the findings of this study are available from the corresponding authors, Yukun Li or Xun Chen, upon reasonable request.

## Supplementary Materials

The supplementary material is available online at https://www.techscience.com/doi/10.32604/or.2026.071536/s1.
