# Supplementary material for: Lactic Acid Drives ESM1 to Attenuate DNA Damage and CD8+ T Cell Infiltration in Cancer
Source: Oncol Res. 2026 Mar 23;34(4):28. doi: 10.32604/or.2026.071536 (PMC13040346; doi:10.32604/or.2026.071536)
Supplement: Supplementary file 1 [file OncolRes-34-71536-s001.doc]

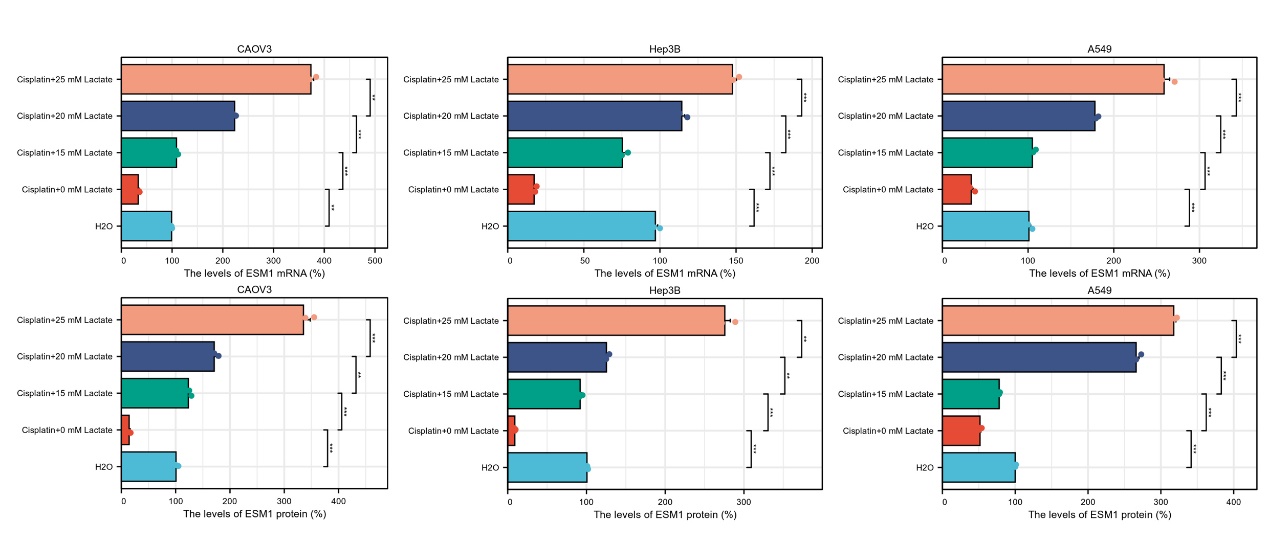


**Supplementary Figure S1. Densitometry quantification for WB and PCR.** The comparative analysis of ESM1 expression employed densitometric evaluation. Upper panels show mRNA profiles via RT-PCR, while lower panels display protein patterns by WB normalized to β-actin. Identical tissue samples ensured direct comparison between mRNA and protein levels. **p < 0.01, ***p < 0.001.


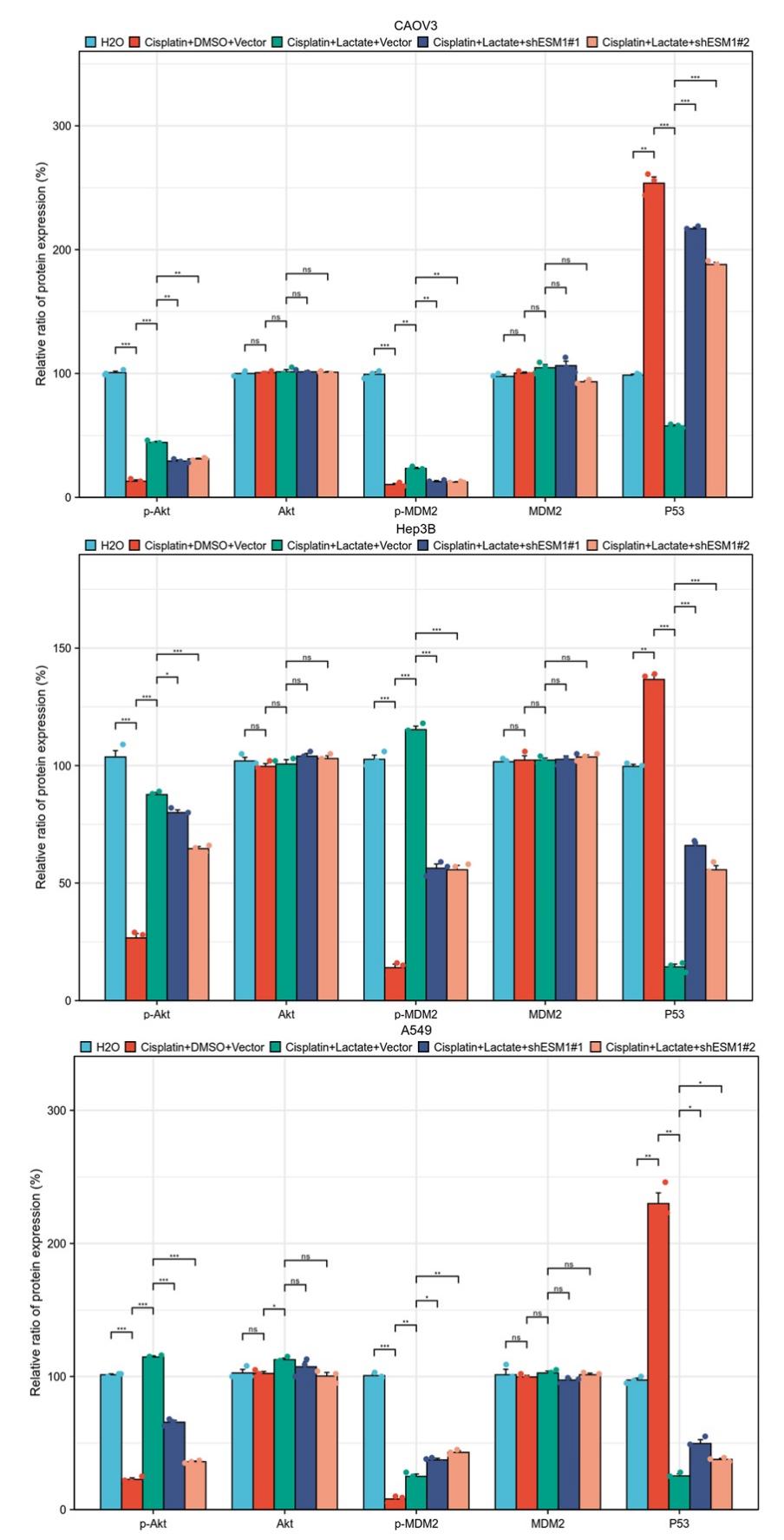


**Supplementary Figure S2. Densitometry quantification for WB.** The comparative analysis of p-Akt1, Akt1, p-MDM2, MDM2, and P53 expression employed densitometric evaluation. ns p > 0.05, *p < 0.05, **p < 0.01, ***p < 0.001.


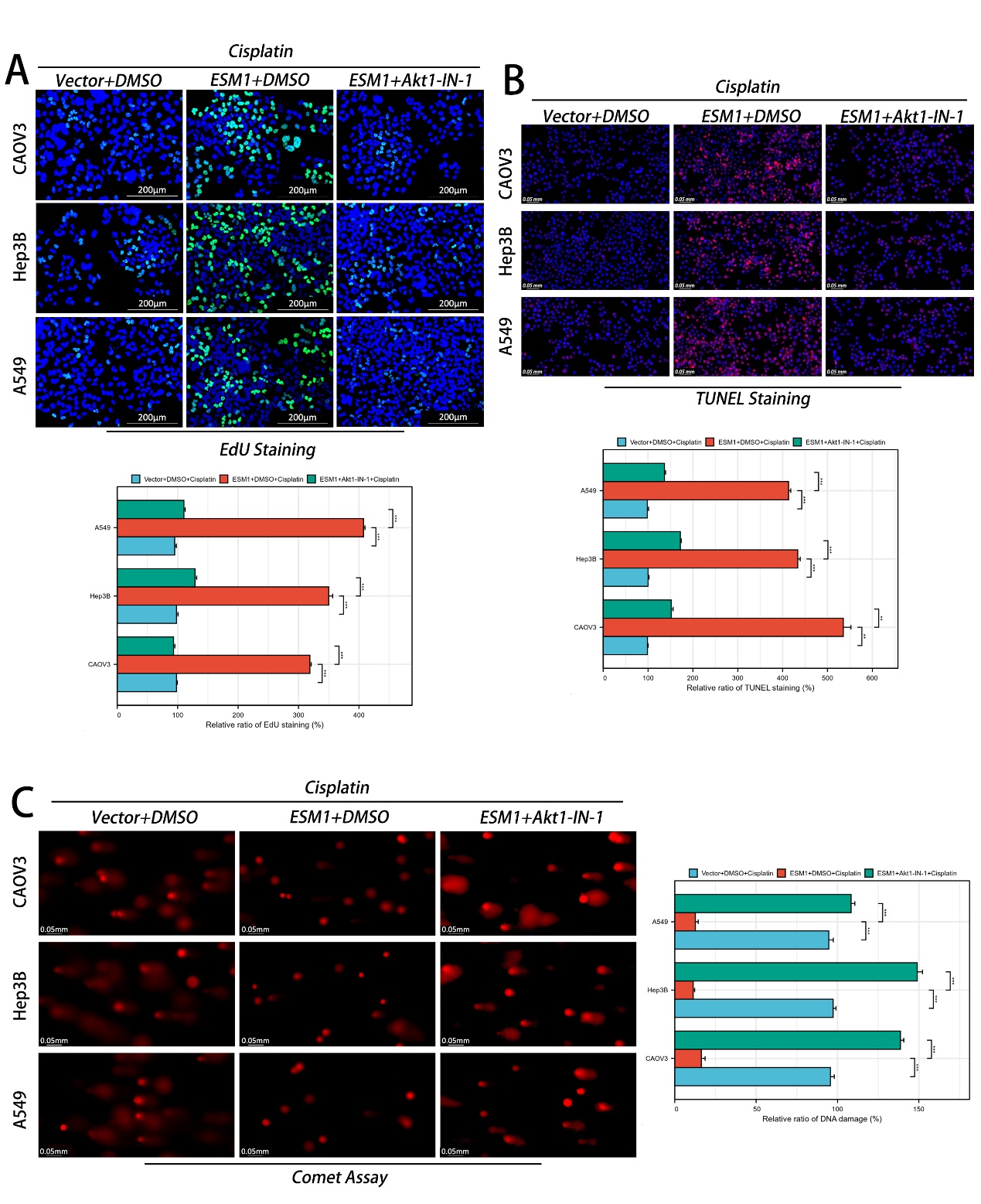


**Supplementary Figure S3. AKT1 inhibitor antagonizes ESM1-induced cell proliferation and apoptosis evasion.** A. EdU staining to confirm the effect of Akt1 inhibitor on ESM1-mediated cell proliferation within cisplatin treatment. B. TUNEL staining to confirm the effect of Akt1 inhibitor on ESM1-mediated cell apoptosis within cisplatin treatment. C. Comet assay to confirm the effect of Akt1 inhibitor on ESM1-mediated cellular DNA damage repair capacity within cisplatin treatment. **p < 0.01, ***p < 0.001.


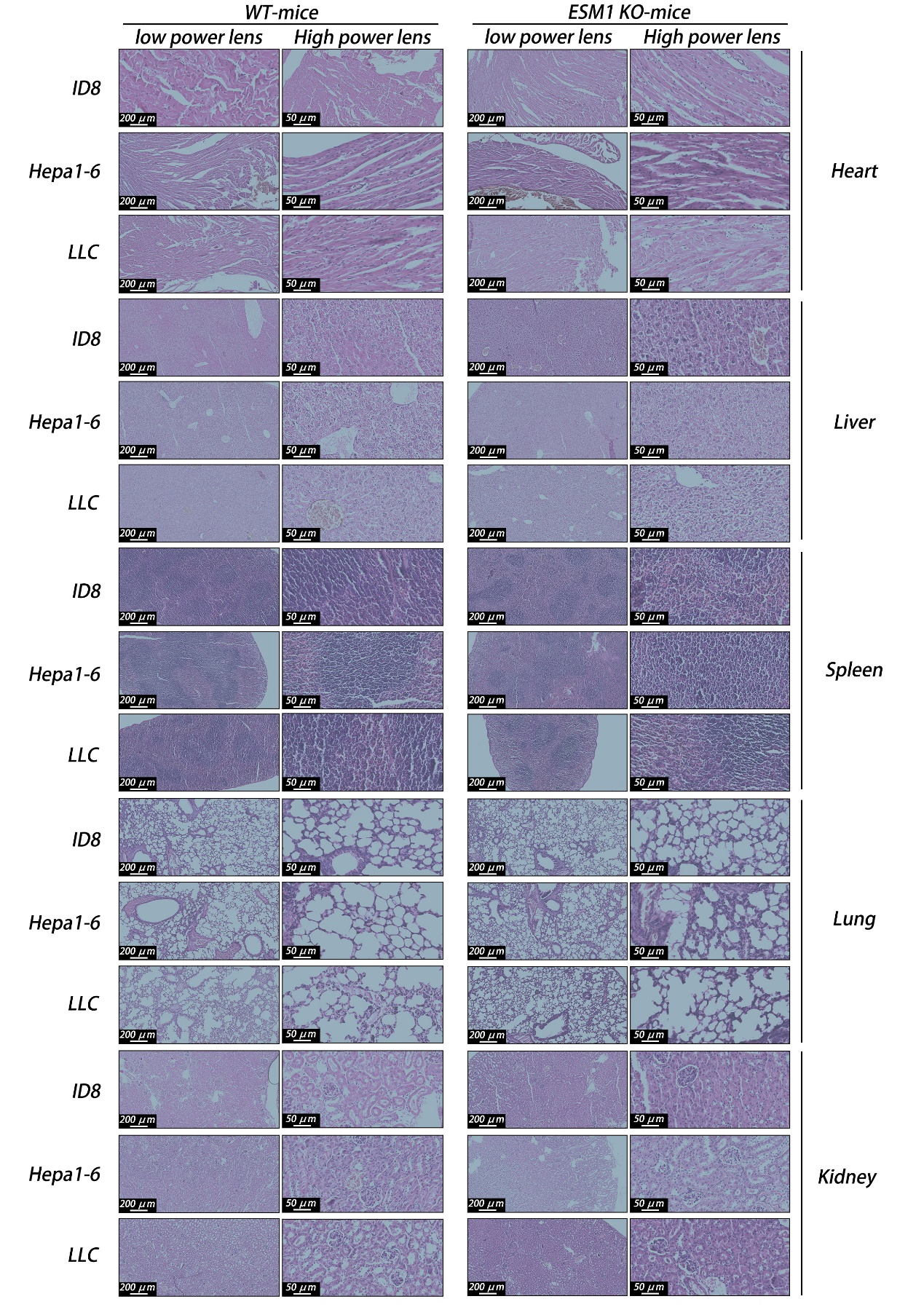


**Supplementary Figure S4. Morphological differences in various organs, including the heart, liver, spleen, lung, and kidney, between ESM1-KO mice and wild-type mice.**
